# SARS-CoV-2-assoziierter Pneumothorax, Pneumomediastinum und Weichteilemphysem. Klinische Implikationen anhand einer Fallserie

**DOI:** 10.1007/s10354-021-00872-4

**Published:** 2021-08-12

**Authors:** Iurii Mykoliuk, Alfred Maier, Jörg Lindenmann, Freyja-Maria Smolle-Jüttner

**Affiliations:** Klinische Abteilung für Thoraxchirurgie und Hyperbare Chirurgie, Medizinische Universität Graz, Auenbruggerplatz 29/3, 8036 Graz, Österreich

**Keywords:** Coronavirus, SARS-CoV‑2, Pneumothorax, Pneumomediastinum, Weichteilemphysem, Coronavirus, SARS-CoV‑2, Pneumothorax, Pneumomediastinum, Soft tissue emphysema

## Abstract

Dass 2019 neu aufgetretene Coronavirus (SARS-CoV-2) bewirkt ein breites Spektrum an Symptomen und Verläufen. Pneumothorax, Pneumomediastinum und Weichteilemphysem sind seltene Komplikationen im Rahmen pulmonaler Beteiligung. Sie entstehen auf Basis der schweren, virusbedingten Lungenveränderungen und werden durch das Erfordernis hoher Beatmungsdrücke aggraviert. Pneumothorax und ektope Luft in Mediastinum und Weichteilen sind damit Indikatoren für gravierende Lungenschäden. Gerade deshalb müssen auch kleine bzw. multipel rekurrierende Pneumothoraxe durch Drainage therapiert werden.

## Abstract (en)

The new coronavirus (SARS-CoV-2) that arose in 2019 causes a wide spectrum of symptoms and different courses of disease. Pneumothorax, pneumomediastinum and soft tissue emphysema are rare complications in patients with pulmonary involvement. They are the sequelae of severe, virus-induced structural changes of the pulmonary architecture. High pressure artificial ventilation aggravates the problem. Hence pneumothorax and ectopic air in soft tissues are indicators of extensive pulmonary damage. Therefore, efforts should be made to treat even very small or multiply recurrent pneumothorax by drainage procedures.

Das neue Coronavirus SARS(„severe acute respiratory syndrom“)-CoV‑2 führt zu einer Vielzahl unterschiedlicher Symptome und klinischer Verläufe [1–5, 12].

Die Verlaufsform mit der höchsten Mortalitätsrate ist die durch SARS-CoV‑2 verursachte Pneumonie. Sie führt in einem hohen Prozentsatz zu adultem akutem Lungenversagen (ARDS), das sowohl durch intravaskuläre Thromben als auch durch Zerstörung der alveolären Strukturen und fibrotischen Umbau der Lunge charakterisiert ist. Wird eine derartige Pneumonie überlebt, sind pulmonale Langzeitfolgen häufig [12]. Im Verlauf einer schweren, SARS-CoV-2-bedingten Pneumonie können Pneumothoraxe, Weichteilemphysem und ein Pneumomediastinum entstehen. Diese seltenen Konstellationen entwickeln sich aus unterschiedlichsten klinischen und radiologischen Ausgangssituationen und sind häufig mit einer schlechten Prognose verbunden [4]. Das gilt insbesondere für das Pneumomediastinum [2, 7, 10, 16].

Die folgende Fallserie zeigt die Variabilität und fehlende Vorhersagbarkeit der Verläufe von durch Pneumothorax, Pneumomediastinum und Weichteilemphysem komplizierter SARS-CoV-2-Pneumonie.

## Fall 1

**Figure 1:**
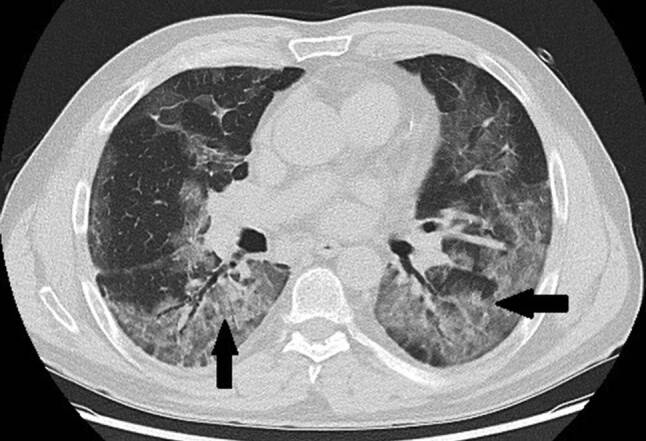

Ein 71-jähriger Patient wurde aufgrund rasch zunehmender respiratorischer Insuffizienz aufgenommen. Das Thorax-CT bei der Aufnahme zeigte bilaterale milchglasartige Verschattungen mit Punctum maximum in den Unterlappen (Abb. 1). Die PCR bestätigte die Infektion mit SARS-CoV‑2. Seit 9 Tagen bestanden Myalgien, Diarrhö und Anosmie. Anamnestisch lag Asthma bronchiale unter Bedarfstherapie vor. Die Kreislaufparameter waren unauffällig, die Temperatur subfebril. Unter Raumluft war die Sauerstoffsättigung 78 %, unter 9 l O_2_/min Maskenatmung 92 %. Die Atemfrequenz war auf 52/min gesteigert. Im Labor waren CRP auf 104 mg/l (Normwert 0–5), Ferritin auf 2295 ng/ml (NW 18–360) und IL 6 auf 133 pg/ml (NW 0–7) erhöht. Nach Einleitung antibiotischer, antimykotischer und antiviraler Therapie mit Ampicillin/Sulbactam, Azithromycin und Posaconazol, sowie Dexamethason, Remdesivir und Aciclovir wurde der Patient an der Intensivstation aufgenommen.

**Figure 2:**
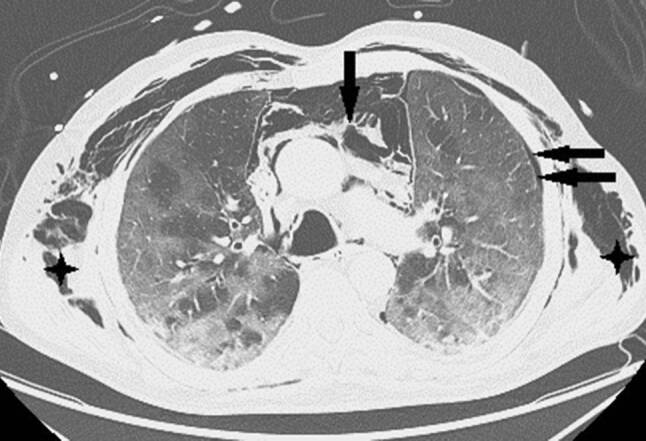

Nach 24 h nichtinvasiver Beatmungsversuche war wegen rasch progredienter, respiratorischer Verschlechterung die Intubation erforderlich. Durch Bauchlagerung und Optimierung der Beatmungsparameter gelang eine vorübergehende Stabilisation der Oxygenierung mit Oxygenationsindizes (OI) bis 150. Am Tag 13 fiel ein rasch progredientes Weichteilemphysem an Hals und linkem Hemithorax auf. Das Thorax-CT zeigte multiple Milchglastrübungen („ground-glass opacity“), Parenchymkonsolidierungen sowie einen schmalen Pneumothorax links mit einer apikalen Spaltbreite bis maximal 15 mm. Zusätzlich bestanden ein ausgedehntes Pneumomediastinum und bilaterales Weichteilemphysem am Thorax (Abb. 2). Nach Anlage einer linksseitigen Thoraxdrainage war das Weichteilemphysem zunächst rückläufig. Wegen dessen erneuter, rascher Zunahme wurden rechts und links je eine weitere Thoraxdrainage gelegt, obwohl rechts kein Pneumothorax nachweisbar war. Danach war das Weichteilemphysem kontinuierlich regredient, die Lungen blieben expandiert. Trotzdem verschlechterte sich die respiratorische Situation unaufhaltsam. Der Patient verstarb nach 17 Tagen invasiver Beatmung (Pressure-Support-Ventilation-Modus, Spitzendrücke 28 mbar, durchschnittlicher „driving pressure“ 17 mbar) und begleitender, intensivmedizinischer Maßnahmen infolge kardiorespiratorischen Versagens.

## Fall 2

Ein 63-jähriger Patient wurde wegen Atembeschwerden in der Notaufnahme vorstellig. Anamnestisch persistierten seit 1 Woche Fieber, Myalgien, Halsschmerzen und zunehmender Husten; 48 h zuvor war die PCR auf SARS-CoV‑2 positiv gewesen. Als Begleiterkrankungen lagen KHK II, Status post NSTEMI mit 2 Drug-eluting-Stents und Asthma bronchiale vor. Der Blutdruck war mit 110/70 mm Hg im leicht hypotonen Bereich, die Körpertemperatur war normal. Unter 8 l O_2_/min Maskenatmung lag die Sauerstoffsättigung bei 89 %. Im Labor waren auf 171 mg/l erhöhtes CRP sowie LDH mit 358 U/l (Normwert [NW] bis 240) auffällig.

**Figure 3:**
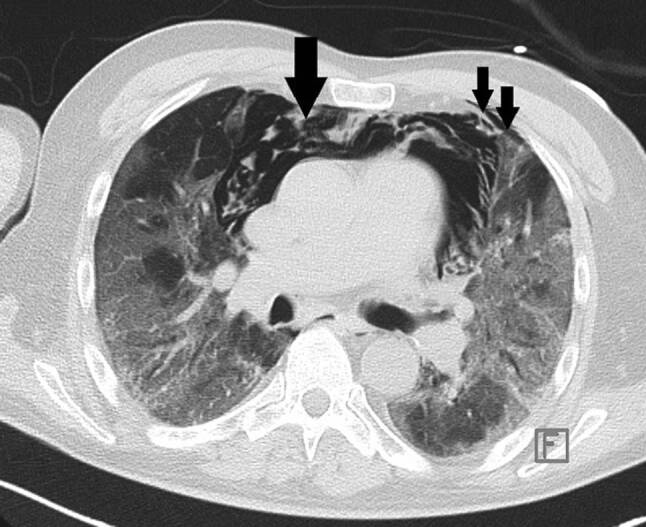

Das Thorax-CT am Aufnahmetag zeigte bilaterale milchglasartige Verschattungen. Antibiose mit Piperacillin/Tazobactam wurde eingeleitet. Unter High-flow-Sauerstofftherapie stabilisierte sich die O_2_-Sättigung bei 92 %. Am Tag 9 entstand eine respiratorische Dekompensation. Nach Stoßtherapie mit 1 g Methylprednisolon über 4 Tage und anschießender Gabe von Prednisolon 50 mg täglich sowie Masken-CPAP-Atmung stabilisierte sich die respiratorische Situation. Das Kontroll-Thorax-CT zeigte Zunahme der Infiltrate, ein Pneumomediastinum und einen minimalen linksseitigen Pneumothorax (Abb. 3). Eine linksseitige Thoraxdrainage wurde gelegt. Am Tag 14 entstand rapide eine respiratorische Globalinsuffizienz, sodass Intubation und in weiterer Folge die Anlage eines Tracheostomas erforderlich wurden.

**Figure 4:**
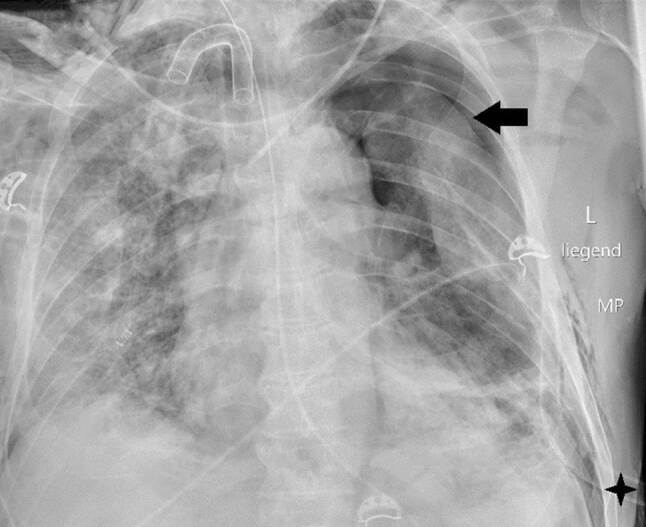

Trotz expandierter Lunge und Regredienz des Mediastinalemphysems persistierten Decarboxylierungs- (pCO_2_ 55–95 mm Hg) und Oxygenierungsprobleme (OI 90–120). Bei liegender Thoraxdrainage entwickelte sich 5 Tage später ein neuerlicher, apikal gekammerter Pneumothorax (Abb. 4), der gezielt mit einer weiteren Drainage versorgt wurde. Nach 16 Tagen Beatmung über den Tubus („dual positive airway pressure“, Spitzendrücke 30 mbar, durchschnittlicher „driving pressure“ 18 mbar) ohne eindeutige Besserungstendenz der respiratorischen Situation und bei weiterer Verschlechterung der Hämodynamik trotz kontinuierlich steigender Katecholamindosen verstarb der Patient am Tag 31 der intensivmedizinischen Behandlung an einer abdominellen Blutungskomplikation.

## Fall 3

**Figure 5:**
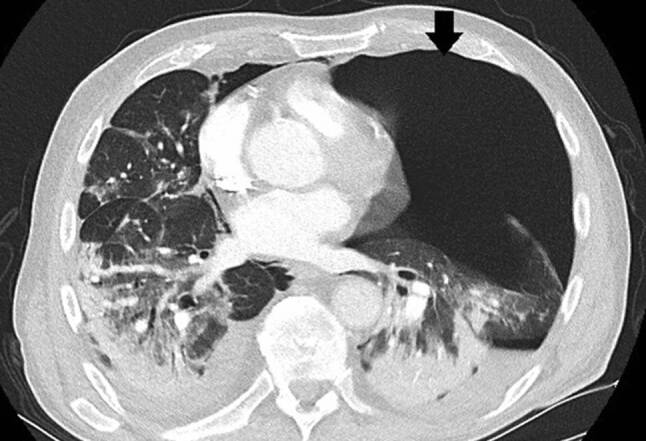

**Figure 6:**
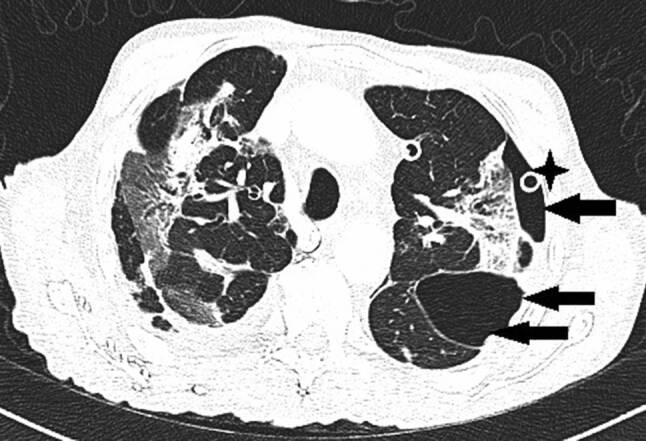

Ein 75-jähriger Patient wurde wegen Thoraxschmerz und Dyspnoe in präkollaptischem Zustandsbild vorgestellt. Vor einigen Tagen habe Husten bestanden, der aber wieder abgeklungen sei. Die PCR auf SARS-CoV‑2 war positiv. Anamnestisch lag arterieller Hypertonus vor. Ein akutes Koronarsyndrom wurde mittels EKG, Echokardiographie und Enzymdiagnostik ausgeschlossen, die Kreislaufparameter waren unauffällig, der Patient afebril. Unter 2 l O_2_/min über Nasenbrille lag die Sauerstoffsättigung bei 94 %. Im Labor bestanden Leukozytose (20,4 G/l), Erhöhung von Ferritin auf 752 ng/ml (NW 18–360) und von IL‑6 auf 29,2 pg/ml (NW 0–7). Das Thorax-CT zum Aufnahmezeitpunkt zeigte SARS-CoV-2-typische Infiltrate sowie einen Pneumothorax links (Abb. 5). Nach Anlage einer Thoraxdrainage links besserte sich die respiratorische Situation rasch. Unter kontinuierlicher O_2_-Gabe (4 l/min) über Nasenbrille erfolgte der Transfer an die Normalstation. Nach Einleitung leitlinienkonformer Therapie mit Antibiotika und Kortikosteroiden waren die Entzündungswerte regredient. Infolge persistenter, pulmopleuraler Fistel reexpandierte die linke Lunge jedoch trotz Anlage eines weiteren Drains nicht. Das Thorax-CT ergab Zeichen inhomogener Lungendestruktion mit bilateralen Fibrosearealen und Pneumatozelen (Abb. 6). Infolge ausgeprägter Kollateralventilation war das auf ein Subsegment fokussierte Setzen eines Ventils nicht möglich. Das Positionieren von mehreren Ventilen schied aus funktionellen Gründen aus.

Eine thorakoskopische Intervention schied ebenfalls aus, da aus anästhesiologisch-beatmungstechnischer Sicht weder die seitengetrennte Ventilation mit Kollabieren der zu operierenden Lunge noch das Setzen eines Pneumothorax möglich waren. Bei der Thorakotomie am Tag 10 fand sich eine ausgeprägte bronchopleurale Fistel, ausgehend von einer rupturierten Pneumatozele.

Das Areal wurde keilreseziert. Nach problemlosem postoperativem Verlauf wurde der Patient am Tag 16 ohne respiratorische Unterstützung nach Hause entlassen.

## Fall 4

Eine 65-jährige Patientin wurde wegen seit einigen Tagen zunehmender Dyspnoe in der Notaufnahme vorstellig. Die PCR auf SARS-CoV‑2 war positiv. Weitere SARS-CoV-2-spezifische Symptome verneinte die Patientin. Anamnestisch lag eine Hypothyreose vor.

**Figure 7:**
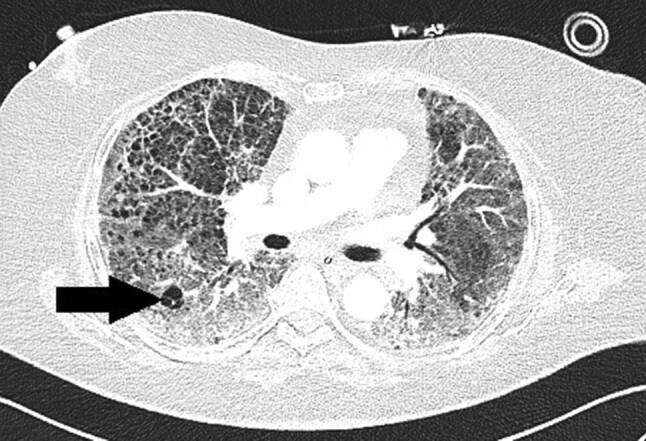

**Figure 8:**
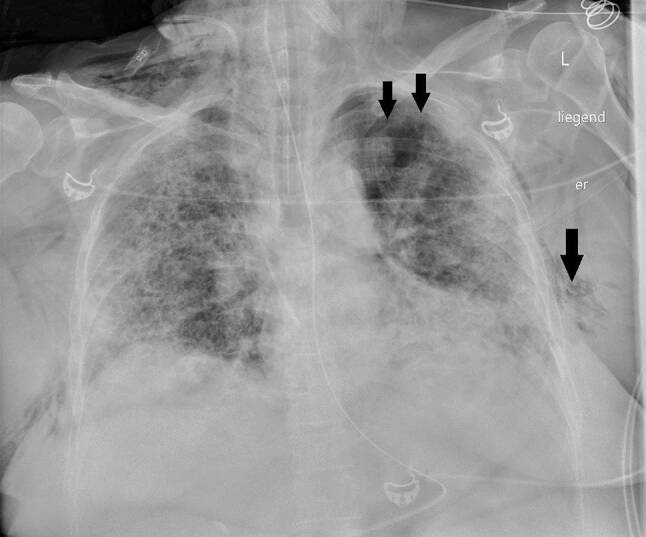

Die Kreislaufparameter waren bis auf leichte Tachykardie von 95/min unauffällig, die Patientin war afebril. Bei erhöhter Atemfrequenz von 28/min betrug die periphere Sauerstoffsättigung 87 % unter 10 l O_2_/min Maskenatmung. Im Labor bestanden Leukozytose von 9,5 G/l sowie Erhöhung von CRP auf 85,7 mg/l, von Ferritin auf 1062 ng/ml und von (IL)‑6 auf 225 pg/ml. In Thorax-CT zeigten sich bilaterale, diffus flächig konfluierende Milchglasverdichtungen (Abb. 7). Nach Einleitung von antibiotischer Therapie und Kortisongabe gelang es zunächst mit nichtinvasiven Beatmungsverfahren, einen Oxygenationsindex von 150 zu halten. Am Tag 6 wurden jedoch Intubation und Beatmung unumgänglich. Am Tag 13 fand sich erstmals ein Weichteilemphysem links thorakal und rechts supraklavikulär. Die Thoraxröntgenaufnahme (Abb. 8) zeigte zusätzlich zum Weichteilemphysem einen schmalen, apikalen Pneumothorax links, der umgehend drainiert wurde. Danach war das Weichteilemphysem regredient. Nach erfolgreichem Weaning von der Beatmung („dual positive airway pressure“, Spitzrendrücke 30 mbar, durchschnittlicher „driving pressure“ 15 mbar für insgesamt 19 Tage) wurde die Patientin am Tag 26 aus der Intensivbehandlung entlassen und am Tag 54 mit residualem, intermittierendem Sauerstoffbedarf von 3 l/min in die externe Rehabilitation transferiert.

## Diskussion

Die SARS-CoV-2-Infektion der unteren Luftwege kann infolge schwerer struktureller Veränderungen des Lungengewebes bzw. von dessen Gefäßsystem zu progredienter respiratorischer Insuffizienz bis hin zum irreversiblen kardiorespiratorischen Versagen führen. Die zusätzliche Entwicklung eines Pneumothorax bzw. eines Mediastinalemphysems kann zur Aggravierung der respiratorischen Einschränkung führen [2, 11, 13]. Pneumothorax und ektope Luft in Mediastinum und Weichteilen sind bei SARS-CoV-2-Infektionen selten. Sie sind Folge virusbedingter Destruktion bzw. Ruptur der Alveolenwände nach Befall der Pneumozyten Typ 1 und Typ 2 [1, 11, 14, 17]. Die intraluminale Luft gelangt aus den zerstörten Alveolen in das Interstitium und migriert zentripetal im peribronchialen und perivaskulären Raum in Richtung Mediastinum. Dieses Phänomen ist unspezifisch und wurde bereits 1944 erstmals beschrieben [8]. Positive Druckbeatmung verstärkt die Lufttransposition durch die Erhöhung des Druckgradienten zwischen Interstitium und intraalveolärem Raum. Über das Mediastinum und den Hals kann Luft auch in die thorakalen Weichteile bzw. in den Pleuraraum gelangen und dort sekundär einen Pneumothorax hervorrufen [2, 7, 16, 18]. Bei SARS-CoV‑2 stellt das Pneumomediastinum offenbar einen prädiktiven Faktor für die Entwicklung des Pneumothorax dar. Etwa 35 % der Patienten mit Pneumomediastinum entwickeln gleichzeitig oder nachfolgend ein Pneumothorax [6].

Die zweite Genese des SARS-CoV-2-assoziierten Pneumothorax ist Luftaustritt aus pleuranahem, destruierten Lungengewebe bzw. Ruptur sekundär inflammationsbedingt entstandener Pneumatozelen [4, 15]. Weichteilemphysem entsteht dabei dann, wenn die intrapleurale Luft über pulmopleurale Adhäsionen Anschluss an das extrapleurale Gewebe hat. Je invasiver die Beatmungstechnik, desto ausgeprägter ist der Luftaustritt [12, 13, 17].

Auch ein schmaler Pneumothorax ist bei beatmeten Patienten mit schwerwiegenden Lungenparenchymveränderungen eine Indikation zur sofortigen Drainage, zumal bereits geringe, durch die extrapleurale Luft bedingte Einschränkung des Lungenvolumens in dieser Situation gravierende Folgen haben kann. Das Pneumomediastinum ist bei geringer Ausprägung häufig selbstlimitierend. Große, mediastinale Luftansammlungen können in Einzelfällen zu Einflussstau auf Vorhofebene führen. In diesen Situationen ist akute Entlastung durch suprajuguläre Eröffnung des Spatium praetracheale indiziert [3]. Durch Anlage von – nötigenfalls bilateraler – Thoraxdrainage gelingt jedoch in den meisten Fällen die Kontrolle ausgeprägter mediastinaler Luftansammlungen [10, 12]. Gleiches gilt für das – per se fast immer harmlose – Weichteilemphysem an Hals oder Thorax, das als klinisch oft augenfälliges Sekundärphänomen eines Pneumothorax bzw. Mediastinalemphysems oft der erste Hinweis auf die intrathorakale Problematik ist.

Zur Inzidenzrate der Entstehung eines Pneumomediastinums und Pneumothorax im Rahmen von SARS-CoV‑2 gibt es noch wenige Daten. Eine Multicenterstudie aus 16 Behandlungszentren in England ergab 60 Fälle von Pneumothorax bei insgesamt 6574 SARS-CoV-2-Aufnahmen, was der Inzidenz von 0,91 % entspricht [9]. Eine ähnliche Inzidenzrate wurde im Zuge des initialen Aufflammens der Pandemie in Wuhan beobachtet [4].

Aus demografischer Sicht scheinen Männer rund 3‑mal so häufig wie Frauen von einem Pneumothorax infolge SARS-CoV‑2 betroffen zu sein. Dies deckt sich mit unseren eigenen Beobachtungen. Eine mögliche Erklärung für die bei Männern höhere Inzidenz könnte die größere Häufigkeit von schweren Verläufen von SARS-CoV‑2 sein. Patienten zwischen dem 60. und 80. Lebensjahr entwickelten am häufigsten Pneumothorax bzw. Mediastinalemphysem. Eine Korrelation zu vorbestehenden Atemwegserkrankungen bzw. Zigarettenkonsum konnte interessanterweise nicht hergestellt werden [9].

Unsere Fallserie zeigt, dass sich Pneumothorax bzw. Mediastinal- und Weichteilemphysem bei SARS-CoV‑2 auf der Basis unterschiedlicher klinisch-radiologischer Verläufe entwickeln können. Bei den Patienten 1 und 2 hatten die typische Beschwerden von SARS-CoV-2 angegeben, bevor sich Dyspnoe entwickelte, die innerhalb kurzer Zeit zu fulminanter, respiratorischer Insuffizienz mit Intubations- und Beatmungsbedarf führte. In beiden Fällen waren die morphologischen Veränderungen an der Lunge ausgeprägt und diffus, der Pneumothorax nur diskret, das Pneumomediastinum dagegen stark ausgeprägt. Ein Emphysem an den Thoraxweichteilen entwickelte sich nur in einem Fall. Beide Patienten verstarben infolge der Viruserkrankung. Bei Patient 3 hatte isolierte diskrete, transiente SARS-CoV-2-Symptomatik in Form von Husten vorgelegen, trotzdem lag zum Aufnahmezeitpunkt eine gravierende, jedoch fokal akzentuierte Lungenparenchymdestruktion vor. Die Ruptur einer Pneumatozele war Ursache für Pneumothorax und eine konservativ nicht beherrschbare, pulmopleurale Fistel, nach deren chirurgischem Verschluss der weitere Verlauf günstig war. Mediastinal- bzw. Weichteilemphysem am Thorax waren nicht zu beobachten. Auch die vierte Patientin zeigte isolierte, pulmonale Symptome mit SARS-CoV-2-typischer, fulminanter Dynamik. Zum Zeitpunkt der Erstdiagnose lagen bereits ausgeprägte, diffuse bilaterale Veränderungen vor. In diesem Fall war das Emphysem in den thorakalen Weichteilen Wegweiser für die Diagnose und Therapie des Pneumothorax. Obwohl die Akutphase überlebt wurde, ist bei der Patientin mit anhaltender Beeinträchtigung zu rechnen.

Inwieweit Pneumothorax und Mediastinalemphysem Indikatoren für ein schlechtes Outcome von SARS-CoV-2-Pneumonien darstellen, bleibt aufgrund der noch ungenügenden Datenlage offen. In jedem Fall sind beide Pathologien mit schweren pulmonalen Verläufen assoziiert [2].

## Schlussfolgerung

Pneumothorax und Pneumomediastinum sind Folgen schwerer Lungenparenchymveränderungen bei SARS-CoV-2-Infektionen in Kombination mit invasiver Beatmung und können sich nach unterschiedlichsten Initialverläufen der Erkrankung entwickeln. Begleitendes Weichteilemphysem an Hals oder Thorax ist häufig erstes Indiz für die intrathorakale Problematik, die – nötigenfalls multiple – Drainageanlagen erfordert.

## References

[1] Aguiar D (2020). Inside the lungs of COVID-19 disease. Int J Legal Med.

[2] Al-Azzawi M (2020). Spontaneous subcutaneous emphysema and pneumomediastinum in COVID-19 patients: an indicator of poor prognosis?. Am J Case Rep.

[3] Byun CS (2013). Vacuum-assisted closure therapy as an alternative treatment of subcutaneous emphysema. Korean J Thorac Cardiovasc Surg.

[4] Chen N (2020). Epidemiological and clinical characteristics of 99 cases of 2019 novel coronavirus pneumonia in Wuhan, China: a descriptive study. Lancet.

[5] González-Pacheco H (2021). Bilateral spontaneous pneumothorax in SARS-CoV-2 infection: a very rare, life-threatening complication. Am J Emerg Med.

[6] Kangas-Dick A (2021). Clinical characteristics and outcome of pneumomediastinum in patients with COVID-19 pneumonia. J Laparoendosc Adv Surg Tech A.

[7] Kolani S (2020). Spontaneous pneumomediastinum occurring in the SARS-COV-2 infection. IDCases.

[8] Macklin MT, Macklin CC (1944). Malignant interstitial emphysema of the lungs and mediastinum as an important occult complication in many respiratory diseases and other conditions. Medicine.

[9] Martinelli AW (2020). COVID-19 and pneumothorax: a multicentre retrospective case series. Eur Respir J.

[10] Maunder RJ (1984). Subcutaneous and mediastinal emphysema. Arch Intern Med.

[11] Menter T (2020). Postmortem examination of COVID-19 patients reveals diffuse alveolar damage with severe capillary congestion and variegated findings in lungs and other organs suggesting vascular dysfunction. Histopathology.

[12] Murayama S, Gibo S (2014). Spontaneous pneumomediastinum and Macklin effect: overview and appearance on computed tomography. World J Radiol.

[13] Okada M (2014). Diagnosis and treatment of patients with spontaneous pneumomediastinum. Respir Investig.

[14] Pernazza A (2020). Early histologic findings of pulmonary SARS-CoV-2 infection detected in a surgical specimen. Virchows Arch.

[15] Placik DA, Taylor WL, Wnuk NM (2020). Bronchopleural fistula development in the setting of novel therapies for acute respiratory distress syndrome in SARS-CoV-2 pneumonia. Radiol Case Rep.

[16] Quincho-Lopez A, Quincho-Lopez DL, Hurtado-Medina FD (2020). Case report: pneumothorax and pneumomediastinum as uncommon complications of COVID-19 pneumonia – literature review. Am J Trop Med Hyg.

[17] Wali A (2020). Pneumomediastinum following intubation in COVID-19 patients: a case series. Anaesthesia.

[18] Wang W (2020). COVID-19 with spontaneous pneumothorax, pneumomediastinum and subcutaneous emphysema. J Travel Med.

